# A structural and metabolic framework for classifying pre-clinical tuberculosis infection phenotypes using 18F-FDG PET-CT: a prospective cohort analysis following M. tuberculosis exposure

**DOI:** 10.1136/thorax-2024-221470

**Published:** 2024-06-24

**Authors:** Jee Whang Kim, Sonam Vadera, Meedya Sharifpour, Amrita Bajaj, Anver Kamil, Pranabashis Haldar

**Affiliations:** 1 NIHR Leicester Biomedical Research Centre, Department of Respiratory Sciences, University of Leicester, Leicester, UK; 2 Department of Respiratory Medicine, University Hospitals of Leicester NHS Trust, Leicester, UK; 3 Department of Nuclear Medicine, University Hospitals of Leicester NHS Trust, Leicester, UK

**Keywords:** Tuberculosis, Imaging/CT MRI etc

## Abstract

Tuberculosis (TB) control efforts are limited by ineffective characterisation of tuberculosis infection (TBI) —a heterogeneous spectrum of pre-clinical infection states, invisible to tools of routine clinical screening, that are associated with variable risk of progression to TB disease. In this prospective study, we use positron emission tomography-CT (PET-CT) as a high-resolution imaging modality to characterise and classify structural and metabolic features observed in 16 asymptomatic household TB contacts with normal chest radiographs. We identify four feature patterns that associate with distinct clinical and microbiological outcomes, supporting potential utility of PET-CT for objective classification of TBI phenotypes.

## Introduction

 Tuberculosis infection (TBI) is defined by T-cell immunoreactivity to *Mycobacterium tuberculosis* (Mtb) antigens in the absence of clinical, radiological or microbiological evidence of disease.^1^ However clinical tests of host immunoreactivity are unable to characterise the underlying heterogeneity of TBI states that underpin prospective TB risk.^2^ Among recent TB contacts, studies consistently demonstrate less than 10% risk of progression to disease, implying that over 90% of those treated would have otherwise remained disease free.^3^ Furthermore, this low incident TB event rate remains the primary endpoint for studies developing novel TB risk biomarkers or evaluating novel preventive therapies, necessitating recruitment of large prospective cohorts observed for prolonged periods.^4 5^ In the absence of effective risk stratification, TBI studies are therefore prohibitively expensive, logistically challenging and a significant barrier to progress.

Positron emission tomography-CT (PET-CT) is a highly sensitive imaging modality that has shown promise in characterising features of TBI in non-human primate models.^6 7^ We recently performed prospective characterisation of HIV-uninfected, asymptomatic, adult household pulmonary TB contacts with normal chest radiographs, using serial [18F]Fluorodeoxyglucose (FDG) PET-CT and targeted invasive sampling at sites of FDG uptake (online supplemental figure 1). We reported a subgroup, exhibiting static and longitudinal PET-CT features of high TB risk, evidenced by Mtb isolation at sites of FDG uptake and increasing inflammatory burden after 3 months indicative of progressive infection trajectory.^8^

In this study, our aim was to systematically and independently characterise and classify the metabolic (PET) and structural (CT) features observed in the cohort and determine their association with prospective clinical outcome and radiological trajectory.

## Methods

The clinical study design is summarised in online supplemental figure 1. Participants were clinically characterised and had a QuantiFERON-TB Gold Plus (QFT) and PET-CT at baseline and after 3 months. QFT was repeated after 3 months to identify converters associated with evolving adaptive immunity. Invasive sampling was performed with bronchoalveolar lavage and/or endobronchial ultrasound guided transbronchial needle aspiration (EBUS-TBNA) of accessible metabolically active lymph nodes by trained operators as indicated by imaging. Participants with PET-CT progression or microbiological evidence of disease received anti-tuberculous therapy (ATT). The remainder with immunologically confirmed TBI were prospectively observed without TB preventive treatment (TPT) for 12 months. Those declining TPT after 12 months continued to be prospectively observed for a further 12 months.

Anonymised thoracic PET and CT components of imaging were supplied in a random participant and temporal sequence for independent reporting by two senior thoracic PET-CT radiologists (AK, AB) who were blinded to clinical information, aside from exposure to Mtb. For PET analysis, radiologists had access to the corresponding CT for anatomical mapping. Both PET and CT features were categorically classified as positive (abnormal), indeterminate and negative (normal), based on a priori criteria (table 1).^9^ Images with discordant reporting were identified (SV) and reviewed at a panel meeting of the blinded radiologists to establish consensus.

**Table 1:** Classification of PET and CT components of PET-CT scans

	CT component	PET component
Positive	One or more ITLNs with short axis diameter ≥10 mm and/or lung parenchymal abnormalities associated with possible TB*	Intrathoracic lesions with SUVmax ≥5†
Indeterminate	One or more subcentimetre ITLNs and/or non-specific lung changes‡	Intrathoracic lesions with SUVmax <5 but greater than the uptake in the liver
Negative	No structural changes	No FDG uptake exceeding physiological uptake

Assessment of trajectory from baseline was made based on within-subject differences at 3-months months in quantitative metrics, such as nodal size and SUVmax. Changes in SUVmax less than 20% were considered not- significant.

* Lung changes associated with possible TB included consolidation with cavity and upper lobe nodularity.

† The threshold was selected based on findings from the studies of non-human primate models, which reported its association with culturable *M. tuberculosis*.

‡ Non-specific lung changes included isolated tree-in-bud changes, single nodule, and fibrosis.

ITLNintrathoracic lymph nodePETpositron emission tomographySUVmaxmaximum standardized uptake valueTBtuberculosis

## Results

**Figure 1:**
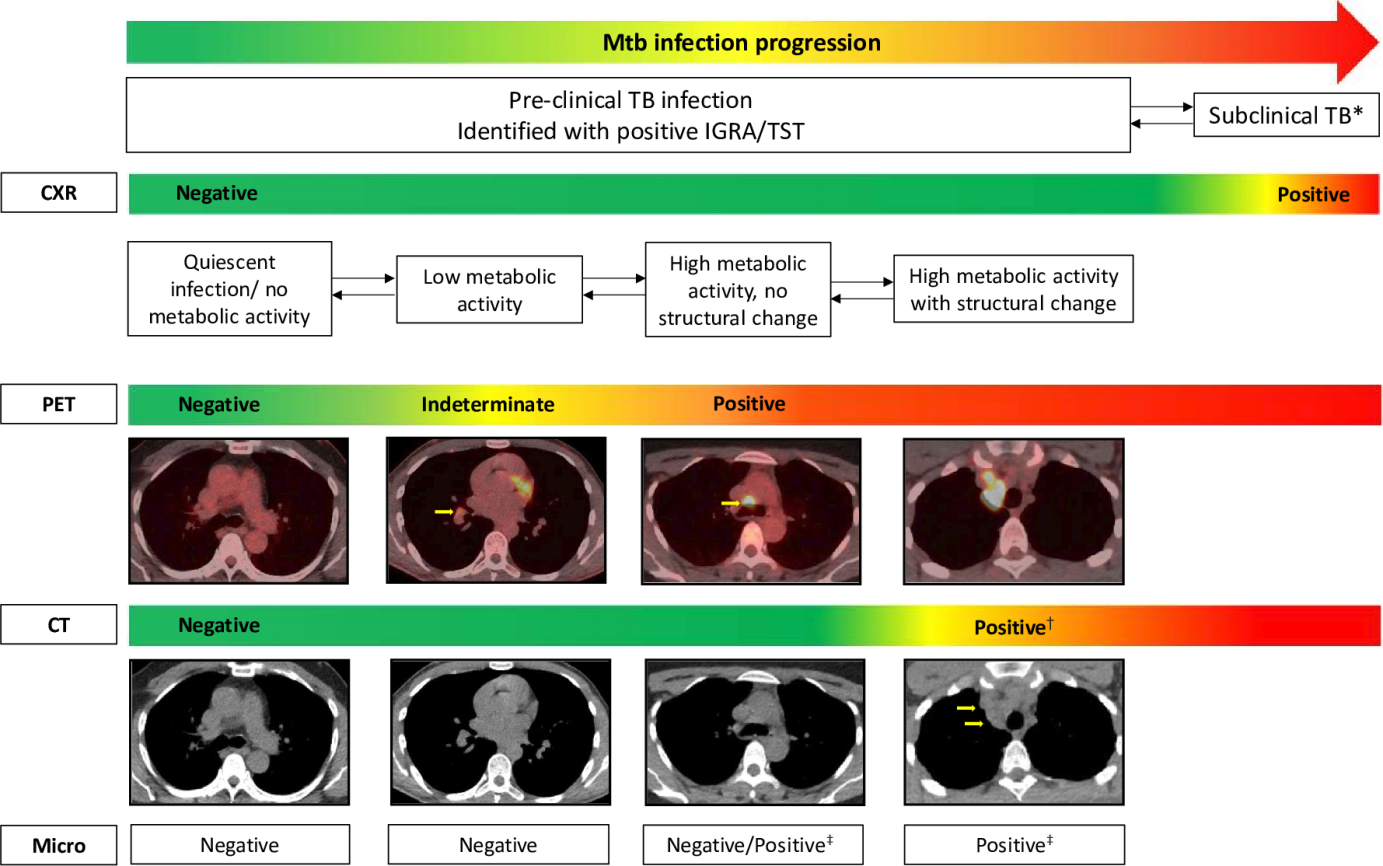
PET-CT-based phenotypes of pre-clinical TB infection. The figure summarises distinct states of TB infection identified by PET-CT in asymptomatic household contacts that precede chest radiographic or sputum-based microbiological detection of disease. *Subclinical TB defined as chest radiographic change and/or positive *Mycobacterium tuberculosis* isolate from sputum. ^†^Structural change on CT that precedes any chest radiographic abnormality. ^‡^*M. tuberculosis* isolated from anatomical sites with increased FDG uptake, in non-expectorating or sputum-negative people. CXR, chest X-ray; FDG, [18F]Fluorodeoxyglucose; IGRA, interferon gamma release assay; Mtb, *Mycobacterium tuberculosis*; PET, positron emission tomography; TB, tuberculosis; TST, tuberculin skin test.

Four of the 20 participating contacts were persistently QFT-negative. One demonstrated indeterminate and static CT lung parenchymal changes of no clinical consequence and all were PET-negative. By definition, this group did not have TBI and is not considered further here. The 16 QFT-positive participants were contacts of nine microbiologically confirmed pulmonary TB index cases (eight smear-positive) and had no reported and/or recorded history of prior TBI. The mean (SD) age of this group was 36.4 (16.5) years, and 14 (87.5%) were foreign-born (13 from Indian subcontinent countries). There was modest agreement (κ_w_=0.371) between baseline PET and CT features. Supported by the outcomes of invasive sampling and follow-up PET-CT, we have categorised four patterns of feature expression and propose evidence for three phenotypic states of metabolically active TBI (table 2, figure 1).

**Table 2:** Participant level PET and CT features at baseline and prospective clinical and radiological outcomes in the 16 QuantiFERON-TB Gold Plus positive participants

Participant number	Baseline PET scan			Baseline CT scan			3-month trajectory of radiological features	Clinical outcomes
	PET outcome	ITLN involvement	Lung involvement	CT outcome	ITLN involvement	Lung involvement		
	Group 1. Baseline positive PET and positive CT							
1*	Positive†	Yes	Yes	Positive	Yes	Indeterminate	N/A‡	ATT
2*	Positive†	Yes	Indeterminate	Positive	Yes	Yes	N/A‡	ATT
3	Positive§	No	Yes	Positive	No	Yes	N/A‡	ATT
4	Positive†	Yes	Yes	Positive	Yes	No	Progressive features on CT and PET††	ATT
	Group 2. Baseline positive PET and indeterminate/negative CT							
5	Positive†	Yes	Yes	Indeterminate	No	Yes	N/A‡	ATT
6*	Positive**	Yes	No	Indeterminate	Yes	No	CT conversion, resolving PET activity	Healthy
7	Positive**	Yes	No	Indeterminate	Yes	No	CT reversion, stable PET activity	Healthy
8	Positive**	Yes	No	Negative	No	No	CT persistently negative, stable PET activity¶	ATT
9	Positive§	Yes	No	Negative	No	No	CT persistently negative, progressive PET activity††	ATT
10	Positive**	Yes	Yes	Negative	No	No	CT remain negative, stable PET activity	Healthy
	Group 3. Baseline indeterminate PET and negative CT							
11*	Indeterminate	Yes	No	Negative	No	No	CT remain negative, PET reversion	Healthy
12	Indeterminate	Yes	No	Negative	No	No	CT remain negative, PET reversion	Healthy
	Group 4. Baseline negative PET and negative CT‡‡							
13	Negative	No	No	Negative	No	No		Healthy
14	Negative	No	No	Negative	No	No		Healthy
15	Negative	No	No	Negative	No	No		Healthy
16	Negative	No	No	Negative	No	No		Healthy

* Demonstrated QFT conversion.

† *M. tuberculosis* identified following invasive samplings.

‡ Received anti-tuberculosis treatment following baseline PET-CT scan.

§ Declined invasive samplings.

¶ Developed symptoms and was diagnosed with ITLN tuberculosis after 2 years.

** No *M. tuberculosis* identified following invasive samplings.

†† Received anti-tuberculosis treatment following 3-month PET-CT scans.

‡‡ Follow upFollow-up PET-CT was not performed.

ATTanti-tuberculosis treatmentITLNintrathoracic lymph nodePETpositron emission tomography

### Positive PET and positive CT (N=4)

This subgroup was characterised by intrathoracic lymph node (ITLN) enlargement (11mm to 16mm in short-axis diameter) with associated FDG uptake on PET in three contacts. Metabolic activity in additional structurally normal nodal stations was observed in two contacts. Lung parenchymal changes were observed on CT in three and on PET in four contacts. Culture isolates of Mtb were obtained in three patients and all four were given ATT. We propose these features represent an advanced state of pre-clinical infection that immediately precedes radiographically visible subclinical TB, in which low-level Mtb is detectable within affected sites identified by high-resolution imaging.

### Positive PET and Indeterminate or negative CT (N=6)

In this subgroup, participants had indeterminate CT features characterised by subcentimetre (8.5–9 mm) ITLNs (n=2) and non-specific upper lobe tree-in-bud nodularity (n=1), with corresponding PET positive metabolic activity, together with evidence of additional PET positivity at structurally normal ITLN stations in all six contacts. Five contacts underwent targeted EBUS-TBNA, with Mtb DNA detected using Xpert MTB/RIF Ultra from a metabolically active structurally normal ITLN in one contact, who received ATT.

The five remaining untreated contacts had follow-up PET-CT. Progressive PET change, characterised by increased FDG uptake in ITLNs and new focal uptake in lung parenchyma, was observed in the contact that had declined EBUS-TBNA, prompting commencement of ATT. Discordance between the trajectory of PET and CT features was observed in two contacts—in one contact, indeterminate CT features resolved with persisting stable PET activity; in the second, CT progression of a subcarinal lymph node from indeterminate (9 mm) to positive (12 mm) was accompanied by resolving PET changes. The remaining two contacts demonstrated stable PET changes within structurally normal ITLNs. At 12 months, all four contacts remained well, declined TPT and continued passive follow-up. One contact with stable 3-month PET changes developed symptoms after 18 months. A further PET-CT showed progressive FDG uptake at the original site of metabolic activity, and sampling confirmed microbiologically positive ITLN TB. In summary, we propose this group represents a phenotype of metabolically active TBI preceding structural pathology that has the potential to evolve, if left untreated.

### Indeterminate PET and negative CT (N=2)

This subgroup was characterised by low-level metabolic activity in structurally normal ITLNs that fully resolved after 3 months. Although more subtle, the changes observed were compatible with a phenotype representing low burden, transient infection that is rapidly controlled and probably cleared.

### Negative PET and negative CT (N=4)

This subgroup did not have follow-up PET-CT scans and remained well after 12 months. It is uncertain whether this represents metabolically quiescent TBI and/or cleared infection with persisting memory T-cell responses. We postulate this group, which accounted for 25% of our TBI cohort, has little or no risk of developing disease, and contributes significantly to low incident TB rates in prospective TBI cohort studies.^10 11^

## Discussion

In this study, we show that addition of PET to CT enables radiographically silent, pre-clinical stages of TBI to be characterised with high sensitivity and specificity, following a history of recent Mtb exposure. Although a semiquantitative measure, we show that SUVmax ≥5 within ITLNs identifies clinically meaningful TBI prior to any structural change. Corresponding lymph node enlargement indicates advancing infection with a higher frequency of detectable microbiological positivity. With 3-month follow-up imaging, we make two important observations. First, discordance between PET and CT can be observed that likely reflects the lag between metabolic trajectory and corresponding evolution or regression of structural change. Second, both progressive and stable 3-month PET changes carry risk of TB progression. This suggests that the fate of infection remains uncertain despite early control of metabolically active TBI, and all persistently PET-positive TBI may therefore be considered to represent potential incipient TB.

Although limited by observations in a small cohort of household contacts, this study suggests the utility of PET-CT to characterise underlying states of TBI. Given the cost and radiation exposure involved, we emphasise PET-CT is not a tool for routine clinical practice but a valuable research tool that provides a phenotypic framework to support mechanistic studies and a new approach for designing smaller and more focused studies for novel TBI biomarker development^12^ and early-phase drug and vaccine evaluation that are critical to the goal of TB elimination.

## supplementary material

10.1136/thorax-2024-221470 online supplemental file 1
