# Supplementary material for: A structural and metabolic framework for classifying pre-clinical tuberculosis infection phenotypes using 18F-FDG PET-CT: a prospective cohort analysis following M. tuberculosis exposure
Source: Thorax. 2024 Jun 24;79(12):e221470. doi: 10.1136/thorax-2024-221470 (PMC11671945; doi:10.1136/thorax-2024-221470)
Supplement: online supplemental file 1 [file thorax-79-12-s001.pdf]

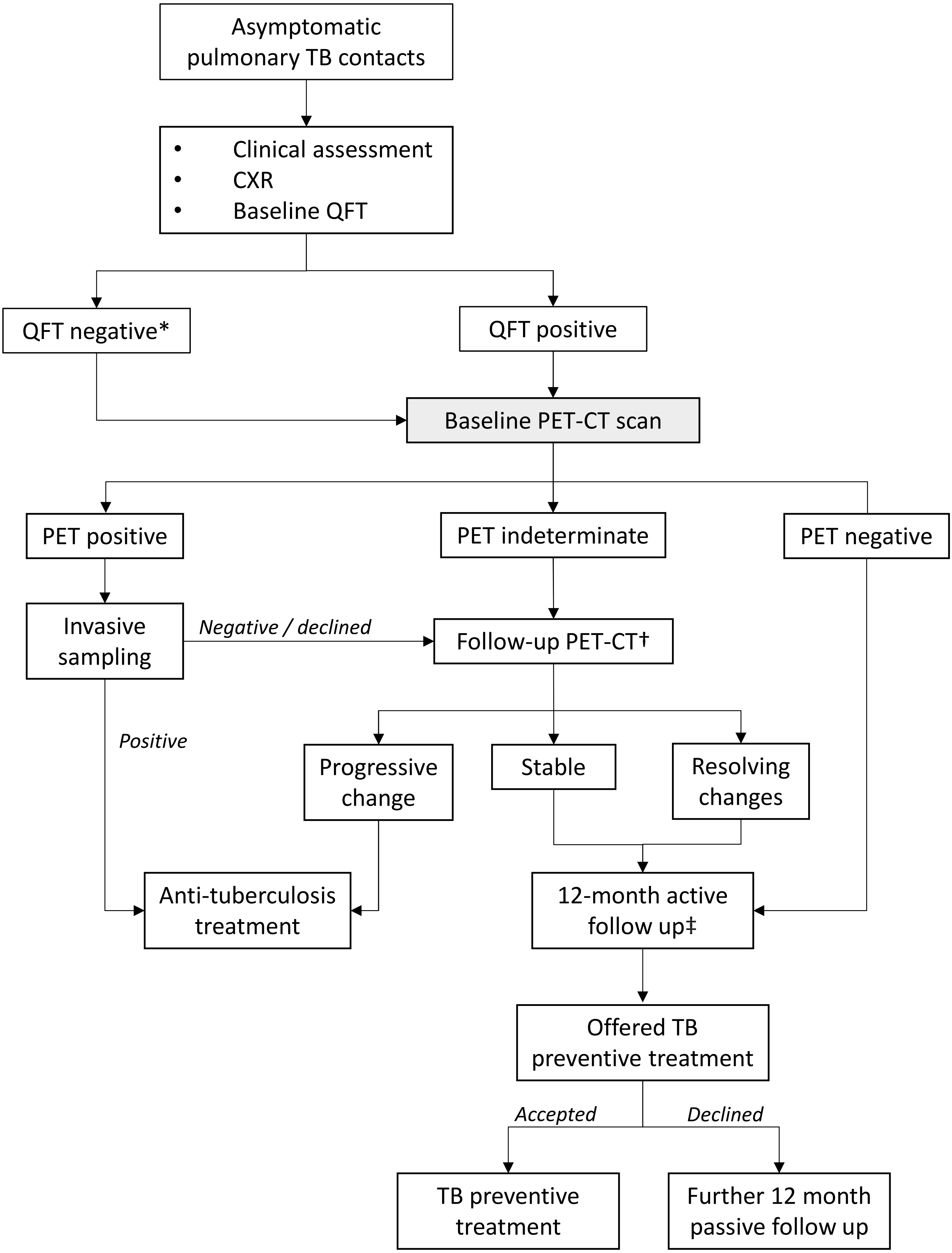

Supplementary figure 1. Study flow diagram

* QFT negative participants had PET-CT scan at baseline only if they remained QFT negative at 3 months.

† QFT was repeated in all participants after 3 months to identify QFT converters

‡ Participants were followed at 3-monthly intervals with symptom questionnaires and CXR.

CXR: chest radiography, PET-CT: positron emission tomography–computed tomography, QFT: QuantiFERON TB Gold-Plus, TB: tuberculosis
